# Highly Sensitive Electrochemical Determination of Butylated Hydroxyanisole in Food Samples Using Electrochemical-Pretreated Three-Dimensional Graphene Electrode Modified with Silica Nanochannel Film

**DOI:** 10.3390/nano14070569

**Published:** 2024-03-25

**Authors:** Chengqing Huang, Shiyue Zhang, Xinying Ma, Fei Yan, Weizhong Tang

**Affiliations:** 1 Guangxi Medical University Cancer Hospital, Guangxi Medical University, Nanning 530021, China; huangchengqing@sr.gxmu.edu.cn; 2 School of Chemistry and Chemical Engineering, Zhejiang Sci-Tech University, Hangzhou 310018, China; 202120104186@mails.zstu.edu.cn (S.Z.); 2023221002036@mails.zstu.edu.cn (X.M.)

**Keywords:** electrochemical sensor, three-dimensional graphene, silica nanochannels, antioxidant, food

## Abstract

The sensitive detection of antioxidants in food is essential for the rational control of their usage and reducing potential health risks. A simple three-dimensional (3D) electrode integrated with an anti-fouling/anti-interference layer possesses great potential for the direct and sensitive electrochemical detection of antioxidants in food samples. In this work, a 3D electrochemical sensor was developed by integrating a 3D graphene electrode (3DG) with vertically ordered mesoporous silica film (VMSF), enabling highly sensitive detection of the common antioxidant, butylated hydroxyanisole (BHA), in food samples. A simple electrochemical polarization was employed to pre-activate the 3DG electrode (p3DG), enhancing its hydrophilicity. Using the p3DG as the supporting electrode, stable modification of VMSF was achieved using the electrochemical assisted self-assembly (EASA) method, without the need for any adhesive agents (VMSF/p3DG). Taking BHA in food as a model analyte, the VMSF/p3DG sensor demonstrated high sensitivity, due to the enrichment by nanochannels, towards BHA. Electrochemical detection of BHA was achieved with a linear range of 0.1 μM to 5 μM and from 5 μM to 150 μM with a low limit of detection (12 nM). Owing to the fouling resistance and anti-interference capabilities of VMSF, the constructed 3D electrochemical sensor can be directly applied for the electrochemical detection of BHA in complex food samples.

## 1. Introduction

The detection of antioxidants in food is crucial to ensuring food quality and safety. As food additives, antioxidants, when used in appropriate quantities, can effectively extend the shelf life of food. However, improper use may pose potential health risks [1,2]. The effective detection of antioxidants can prevent excessive usage or improper combinations, thereby preserving food quality and fostering the healthy advancement of the food industry. Butylated hydroxyanisole (BHA) is a commonly used antioxidant, added to various foods such as fats, fried foods, bread, cereals, candies, and meat products [3,4]. However, excessive intake of BHA may lead to health issues. Research indicates that prolonged high doses of BHA intake may be associated with certain chronic diseases and health problems, including potential impacts on the liver, kidneys, and immune system. Additionally, BHA may carry a certain risk of carcinogenicity. The maximum level of BHA is 100 mg/Kg [5]. Therefore, sensitive detection of BHA in food is essential for the rational control of its usage and reducing potential health risks.

Currently, the main approaches for BHA detection are chromatographic methods, primarily including high-performance liquid chromatography (HPLC) and gas chromatography-mass spectrometry (GC-MS) [6,7]. However, while HPLC offers high accuracy and resolution, it demands relatively advanced instruments and techniques, making the operation intricate. GC-MS holds an advantage in analyzing complex mixtures but also requires meticulous sample preparation and handling. In recent years, electrochemical methods have gained significant attention in the field of BHA detection. Electrochemical techniques possess advantages such as high sensitivity, rapid response, and simple instrumentation [8,9,10,11,12]. For instance, utilizing electrochemical methods like cyclic voltammetry (CV) and linear sweep voltammetry (LSV), specific current responses can be generated during the reduction or oxidation processes of BHA, enabling quantification [5,13]. However, during food sample testing, electrodes are susceptible to contamination by large molecules such as proteins and starch, as well as interference from coexisting electroactive substances (e.g., dopamine (DA) and ascorbic acid (AA)). This significantly impacts the sensitivity and accuracy of the detection. Thus, the development of simple and sensitive electrochemical sensors for the direct electrochemical detection of BHA in food samples without tedious pretreatment is crucial.

Three-dimensional (3D) electrodes have tremendous potential in the construction of electrochemical sensors, as they can significantly enhance sensitivity and response speed [14,15]. Traditional two-dimensional (2D) electrode surfaces are generally flat, with a relatively small surface area, limiting their sensitivity and response speed. In contrast, 3D electrodes have a unique structure with a larger surface area, effectively increasing the density of active sites and significantly enhancing sensor sensitivity. Additionally, the porous structure of 3D electrodes also improves the transfer rate of protons and electrons, enhancing the sensor’s response speed and kinetic performance. Among them, 3D graphene (3DG) materials have garnered attention as carbon materials with unique structures and properties [16,17,18,19]. 3DG is typically formed by arranging, crossing, or stacking graphene layers to create a 3D network structure. This structure imparts excellent electrical and thermal conductivity while retaining outstanding properties such as the light weight and high strength inherent in graphene. For instance, 3DG grown through chemical vapor deposition (CVD) involves decomposing carbon source gases (e.g., methane or ethylene) into carbon atoms in a high-temperature inert atmosphere [20]. These atoms then deposit layer by layer on the surface of a metal catalyst, forming a multi-layered 3DG structure. In addition, 3DG produced in this way exhibits a high surface area, rich porous structure, and excellent conductivity. However, this 3DG has an almost defect-free sp^2^ structure, showing high hydrophobicity and weak chemical modifiability [21,22]. Thus, improving the hydrophilicity of 3DG is crucial for the fabrication of electrochemical sensors. For instance, 3DG can be modified by Prussian blue-gold nanoparticles/polydopamine-mercaptobenzoboric acid (PDA-MPBA/PB-AuNPs) or ceria nanoparticles (CeO_2_) or polydopamine-thionine (PDA-TH) for non-enzymatic and reagentless electrochemical detection of glucose [23] or hydrogen peroxide [24,25]. It can also be modified through nitrogen doping for the detection of dopamine [26]. In addition, after modification with antibodies, 3DG-based immunosensors can be applied for sensitive detection of tumor biomarkers [27]. Thus, improving the hydrophilicity as well as electrochemical activity of 3DG is highly desirable for advancing the development of high-performance 3D electrodes.

Enhancing the anti-fouling/anti-interference ability of the electrode is a key issue for the analysis of complex samples [28,29,30,31,32,33,34,35]. A selectively permeable film on the electrode surface, such as an ion-selective membrane, molecular sieve membrane, etc., can block or exclude irrelevant substances from the analysis [36,37,38]. Amongst these, introducing a molecular sieve film on the electrode surface is flexible and effective. For instance, vertically ordered mesoporous silica film (VMSF) has garnered attention due to its unique structure [39,40,41,42]. VMSF is composed of a vertical array of nanochannels perpendicular to the electrode, uniform nanochannel sizes (typically 2–3 nm in diameter), high pore density (up to 10^12^/cm^2^), adjustable thickness (usually 50–200 nm), and good chemical stability [43,44,45]. Compared to the morphologies of other porous silica materials, these characteristics provide VMSF with rapid mass transfer capabilities and size and electrostatic sieving capabilities at the molecular level [46,47,48,49,50]. For instance, it has been proven that the monomer of microperoxidase-11 (MP-11, Mw 1861, size: 1.1 × 1.7 × 3.3 nm), a heme-containing peptide with only 11 amine acids, can enter the nanochannels of VMSF through adsorption. When MP-11 aggregates, the resulting aggregates, due to their increased size, are unable to enter the nanochannels [51]. Thus, VMSF can effectively hinder particles and large molecules (e.g., proteins, DNA, polysaccharides) through steric hindrance [52,53,54]. At the same time, the silanol groups in the silica structure of VMSF have a low ionization constant (p*K*_a_~2). Thus, the silanol groups ionize in conventional solution media, leading to negatively charged surface for VMSF nanochannels. Consequently, VMSF exhibits electrostatic attraction towards positively charged small molecules and electrostatic repulsion against negatively charged small molecules (e.g., redox small molecules such as AA or UA) [55]. Thus, VMSF-modified electrodes demonstrate outstanding anti-fouling and anti-interference, offering unique advantages in direct analysis of complex samples without the need for sample pretreatment. However, carbon-based electrodes cannot directly stabilize the adhesion of VMSF. It has been proven that introducing an adhesive molecule/layer or pre-treating the electrodes can achieve stable binding of VMSF on carbon electrodes. For instance, an organic siloxane, 3-aminopropyltriethoxysilane (APTES), was electro-grafted as an adhesive to enhance the adhesion between VMSF and 3DG [56]. Nevertheless, the non-conductive nature of this adhesive layer may lead to the passivation of the electrode’s electrochemical activity. Reduced graphene oxide (rGO) was also employed as a conductive adhesive layer, successfully growing VMSF on the GCE for the detection of small drugs (acetaminophen (APAP), norepinephrine (NE)) or bioactive molecules (tryptophan (Trp), guanine (G)) [57]. However, the drop-coated rGO layer is prone to unevenness. On the other hand, 3DG can achieve stable binding with VMSF by introducing oxygen-containing groups on the surface through oxygen plasma treatment, allowing for the electrochemical detection of UA [14]. However, plasma treatment requires complex instruments. Integrating VMSF with a 3DG electrode through a simple process holds promise for creating high-performance modified electrodes for the direct and rapid electrochemical detection of BHA in complex food samples.

**Figure 1:**
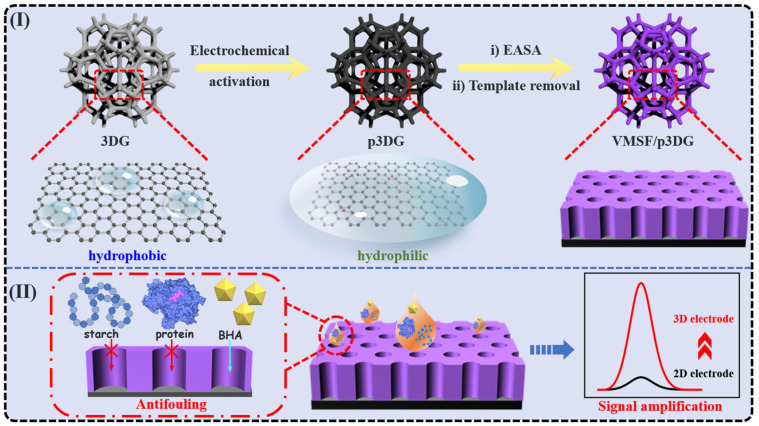
Schematic illustration for (**I**) the fabrication of the VMSF/p3DG sensor and (**II**) the corresponding anti-fouling (the dashed-line box on the left) and signal amplification (the solid-line box on the right) for BHA detection (the illustration in the middle).

In this study, a convenient method was employed to integrate the 3DG electrode with VMSF, constructing a high-performance 3D electrochemical sensor for direct electrochemical detection of BHA in food samples. As illustrated in Figure 1, a simple electrochemical polarization method was employed to enhance the hydrophilicity and electrochemical activity of 3DG. Specifically, 3DG underwent electrochemical polarization by applying a high positive potential for anodic oxidation, followed by a negative potential for cathodic reduction. This process was performed using the commonly used phosphate-buffered saline (PBS), requiring no complex chemical reagents and cumbersome operations. Using the pre-activated 3DG (p3DG) as the supporting electrode, stable modification of VMSF on the electrode surface was achieved through electrochemical-assisted self-assembly (EASA) without the use of any adhesive agents. Both p3DG and VMSF contributed to enhancing the electrochemical signal of butylated hydroxyanisole (BHA), demonstrating a dual signal amplification. Combining the excellent anti-fouling and anti-interference capabilities of VMSF, the developed 3D electrochemical sensor can be directly used for the electrochemical detection of BHA in complex food samples.

## 2. Materials and Methods

### 2.1. Chemicals and Materials

Sodium chloride (NaCl), potassium chloride (KCl), calcium chloride (CaCl_2_), dopamine (DA), ascorbic acid (AA), glucose (Glu), starch, butylated hydroxyanisole (BHA), disodium hydrogen phosphate dodecahydrate (Na_2_HPO_4_·12H_2_O), 1-butyl-3-methylimidazolium hexafluorophosphate (BMIMPF_6_), tetraethyl orthosilicate (TEOS, 98%), and potassium hydrogen phthalate (KHP) were all purchased from Aladdin Reagent Co., Ltd. (Shanghai, China). Magnesium sulfate (MgSO_4_), sodium nitrate (NaNO_3_), sodium chloride (NaCl), potassium ferricyanide (K_3_[Fe(CN)_6_]), acetonitrile (99.9%), sodium dihydrogen phosphate (NaH_2_PO_4_·2H_2_O), and potassium ferricyanide (K_3_[Fe(CN)_6_], 99.5%) were all obtained from McLane Biochemical Technology Co., Ltd. (Shanghai, China). Cetyltrimethylammonium bromide (CTAB, 99%) was purchased from Kermel Chemical Reagent Co., Ltd. (Tianjin, China). Hexachlororuthenate(III) chloride (Ru(NH_3_)_6_Cl_3_) was acquired from Sigma-Aldrich Corporation. Hydrochloric acid (HCl) was obtained from Shuanglin Chemical Reagent Co., Ltd. (Hangzhou, China). The electro-optical glass substrate with calcium-sodium borosilicate glass was sourced from Kaiwei Optoelectronic Technology Co., Ltd. (Zhuhai, China). All chemicals used in the experiments were of analytical grade and were not subjected to further treatment. PBS with different pH was adjusted using HCl (3 M). The aqueous solutions used in the experiments were prepared using ultrapure water (18.2 MΩ·cm).

### 2.2. Characteriaztions and Instrumentations

X-ray photoelectron spectroscopy (XPS) measurements were conducted using the PHI5300 instrument (Perkin Elmer, Waltham, MA, USA) with a Mg Kα excitation source. Scanning electron microscope (SEM) tests were performed using the SU8100 instrument (Hitachi, Tokyo, Japan), with an acceleration voltage of 10 kV. Contact angles were measured using the dynamic contact angle analyzer (DCA-322, Themocahn, Waltham, MA, USA). Electrochemical tests, including cyclic voltammetry (CV) and linear sweep voltammetry (LSV), were carried out on the CHI660e electrochemical workstation (Shanghai Chenhua Instrument Co., Ltd., Shanghai, China). A conventional three-electrode system was employed. Specifically, 3DG, p3DG, or VMSF/p3DG acted as the working electrode (electrode size: 0.5 cm × 0.5 cm). Platinum wire or platinum foil was used as the counter electrode. For non-aqueous electrolyte, the reference electrode used was Ag/Ag^+^ (with 10 mM Ag^+^ in acetonitrile solution as the internal reference solution). In the case of aqueous electrolytes, the reference electrode was Ag/AgCl (with a saturated KCl solution as the internal reference solution).

### 2.3. Preparation of p3DG

The preparation of the 3DG electrode was carried out following the method reported in reference [58]. Briefly, 3DG was synthesized through chemical vapor deposition (CVD, Lindberg Blue M Tube furnace, Thermo Scientific, USA) at 1000 °C using foam nickel as a template and ethanol as the precursor under an H_2_ (25 sccm)/Ar (50 sccm) environment. Subsequently, the obtained 3DG foam containing the nickel template was immersed in a 3 M HCl solution at 80 °C for 24 h to remove the nickel template. Then, the cut piece of 3DG foam (0.5 cm × 0.5 cm) was fixed to a glass slide using silicone gel, and an electrical connection between the 3DG and a copper wire was established using conductive silver paste. Finally, the connection point of the conductive silver paste and the copper wire was sealed with silicone gel, resulting in the 3DG electrode.

Electrochemical activation of the 3DG electrode was achieved through electrochemical polarization involving both anodic oxidation and cathodic reduction. A solution of acetonitrile containing 10% (*v*/*v*) BMIMPF_6_ ionic liquid served as the electrolyte for anodic oxidation. For anodic oxidation, the 3DG electrode functioned as the working electrode, and a constant voltage of +5 V was applied for 100 s. Subsequently, cathodic reduction was performed using the obtained electrode as the working electrode, applying a constant voltage of −1.0 V in phosphate buffer solution (PBS, 0.1 M, pH 6) for 300 s. This process led to the formation of the p3DG electrode.

### 2.4. Preparation of VMSF/p3DG Electrode

The growth of VMSF on the p3DG electrode was achieved through the EASA method [59]. Specifically, a precursor solution for VMSF growth was obtained by stirring a mixture solution containing ethanol (20 mL), NaNO_3_ (20 mL, 0.1 M, pH = 2.6), TEOS (2.833 g), and CTAB (1.585 g) for 2.5 h. Then, the clean p3DG electrode was immersed in the precursor solution as the working electrode, and a constant current of −350 μA was applied for 10 s. Subsequently, the electrode was immediately taken out and thoroughly rinsed with ultrapure water. Finally, it was aged overnight at 80 °C to obtain an electrode with surfactant micelles (SM), denoted as SM@VMSF/p3DG. The SM@VMSF/p3DG electrode was then stirred in a 0.1 M HCl-ethanol solution for 5 min to remove the micelles, resulting in the VMSF/p3DG electrode.

To compare the effect of the dimensions of the underlying electrode, a simple procedure was performed on the GCE to construct a two-dimensional (2D) sensor. Briefly, the GCE was polished with 0.3 μm and 0.05 μm aluminum oxide powder, followed with ultrasonic washing in ethanol and ultrapure water, respectively. The newly polished GCE was then pre-activated using electrochemical polarization. Using PBS (0.1 M, pH 5) as the electrolyte solution, the GCE was anodized at a constant voltage of +1.8 V for 300 s and subject to cathodic reduction at −1.0 V for 60 s to obtain an electrically activated electrode (pGCE). VMSF was prepared by the constant current growth method (current density −0.74 mA/cm^2^) with a duration of 10 s. The obtained electrode was thoroughly cleaned with ultrapure water and aged at 80 °C overnight to obtain a VMSF-modified pGCE containing the SM template, denoted as SM@VMSF/pGCE. SM was removed by stirring in 0.1 M HCl-ethanol solution for 5 min to obtain VMSF/pGCE.

### 2.5. Electrochemical Determination of BHA Using the VMSF/p3DG Electrode

PBS (0.1 M, pH 5) was employed as the electrolyte for electrochemical determination of BHA. The VMSF/p3DG electrode was applied as the working electrode, and the peak current when BHA was electrochemically oxidated on the electrode was measured using linear sweep voltammetry (LSV) [60,61]. Briefly, when different concentrations of BHA were added to the electrolyte, the LSV curves of BHA on the VMSF/p3DG were measured. For real sample analysis, the content of BHA in edible oil (Golden Guan, Taiyuan, China) or coffee (Nestle, Glendale, AZ, USA) was determined using the standard addition method. Specifically, for the detection of BHA in edible oil, edible oil solution (100 mg/mL) in the absence or presence of spiked BHA was prepared using ethanol as the solvent. Subsequently, the obtained solution was diluted 50 times with PBS buffer (0.1 M, pH 5) before testing. In the case of detecting BHA in coffee, the coffee solution (100 mg/mL, prepared by diluting coffee powder in hot ultrapure water, followed by cooling) without or with spiked BHA was diluted 50 times with PBS buffer (0.1 M, pH 5) before measurement.

## 3. Results and Discussion

### 3.1. Characterization of 3DG and p3DG

In this work, 3DG was employed as the supporting electrode. However, 3DG has an almost defect-free sp^2^ structure, showing high hydrophobicity [62,63]. Thus, the detection performance of the fabricated electrochemical sensor often deteriorates due to low wettability and high non-specific adsorption. Additionally, carbon-based electrodes cannot directly stabilize the adhesion of VMSF. Figure 1 shows the VMSF/p3DG sensor and the corresponding anti-fouling and signal amplification characteristics for BHA detection. Electrochemical polarization was employed as a simple and environmentally friendly method to improve the wettability and the electrochemical activity of 3DG. The electrochemical polarization involves anodic oxidation (+5 V) under high potential and cathodic reduction under low potential (−1 V). If the anodic oxidation process takes place in an aqueous solution, water will undergo significant electrolysis under the applied potential, generating a large amount of gas, which can disrupt the structure of 3DG. Thus, anodic oxidation is carried out in organic solvents (acetonitrile) containing ionic liquid (BMIMPF_6_, 10%) to avoid bubble formation during the oxidation process. The subsequent cathodic reduction takes place in an aqueous PBS. Then, a VMSF anti-fouling layer is directly integrated into the pretreated 3DG electrode (p3DG).

**Figure 2:**
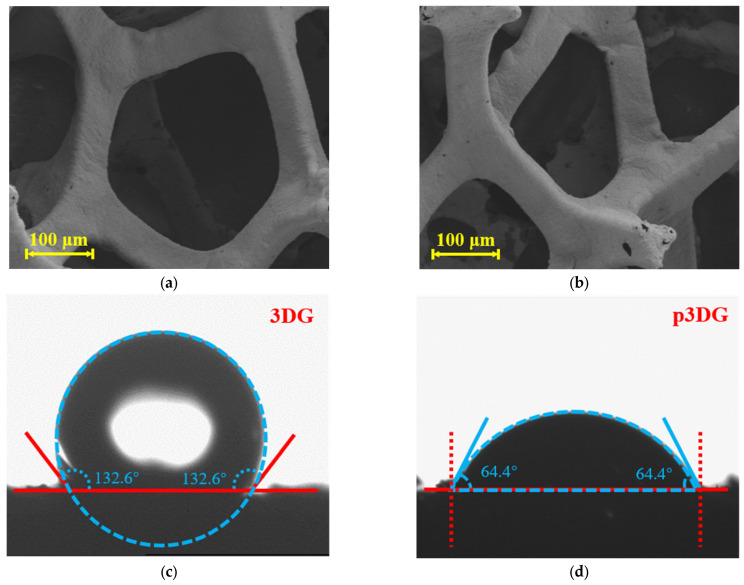
SEM images of (**a**) 3DG and (**b**) p3DG. Contact angle image of (**c**) 3DG and (**d**) p3DG.

The morphology of 3DG and p3DG was characterized through scanning electron microscopy (SEM). As shown in Figure 2a, 3DG exhibits a large-pore graphene grid structure with a smooth and intact surface. After electrochemical activation, p3DG retains the grid structure of 3DG but shows a small number of surface defects (Figure 2b). Changes in the wettability of 3DG before and after polarization were assessed through contact angle measurements. As illustrated in Figure 2c, the contact angle of 3DG is 132.6°, demonstrating high hydrophobicity. In contrast, the contact angle of p3DG after electrochemical activation is 64.4°, indicating significantly enhanced hydrophilicity (Figure 2d). As VMSF is nano-scale film, the obtained VMSF/p3DG also demonstrates the 3D macroporous structure (Figure S1 in Supporting Information).

**Figure 3:**
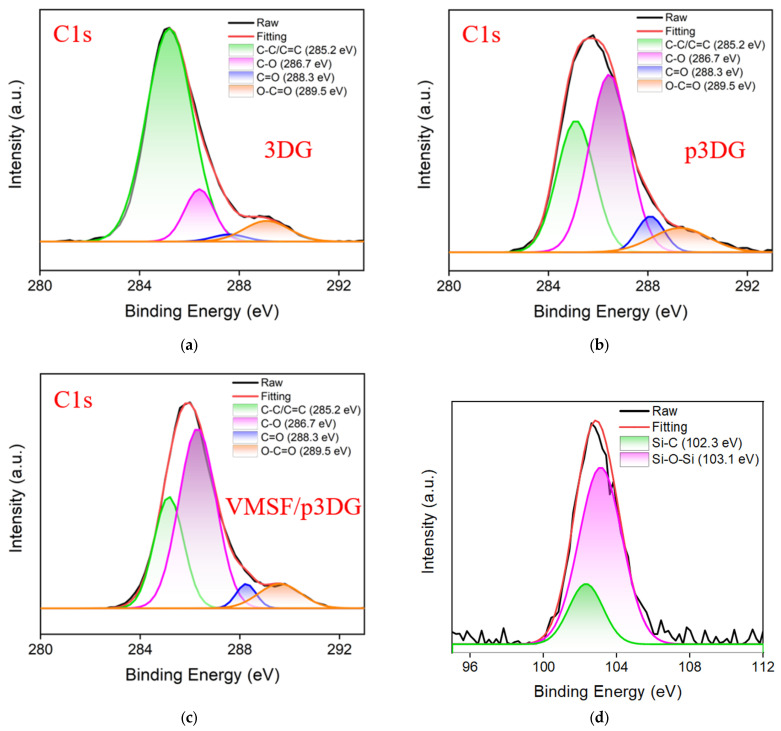
High-resolution C1s spectra of (**a**) 3DG, (**b**) p3DG, and (**c**) VMSF/p3DG. High-resolution Si2p spectrum of (**d**) VMSF/p3DG.

The chemical structure of the 3DG, p3DG, and VMSF/p3DG electrodes was analyzed through XPS. As depicted in Figure 3a, 3DG exhibits a high content of C-C/C=C (285.2 eV), indicating abundant sp^2^ carbon atoms on 3DG. A small amount of oxygen functional groups might originate from water or oxygen in the air. Figure 3b shows the high-resolution C1s spectrum of p3DG, revealing a decrease in C-C/C=C content compared to 3DG while the content of C-O (286.7 eV), C=O (288.3 eV), and O-C=O (289.5 eV) increases, suggesting that the surface of p3DG is rich in oxygen functional groups. This is attributed to the active oxygen radicals generated during the polarization of 3DG, which oxidize and etch the sp^2^-conjugated carbon on the surface, resulting in abundant edge carbon, defects, and oxygen functional groups. After the growth of VMSF, as shown in Figure 3c, the C1s spectrum of VMSF/p3DG is similar to that of p3DG, indicating that the growth of VMSF does not affect the chemical structure of the underlying electrode. Compared to the negligible background Si signal on p3DG, the Si content on VMSF/p3DG significantly increases (7.11%). This is attributed to the silica structure of VMSF (Figure S2 in Supporting Information). The corresponding Si2p spectrum further confirms the successful growth of VMSF on p3DG (Figure 3d).

### 3.2. Charcterization of VMSF on p3DG

**Figure 4:**
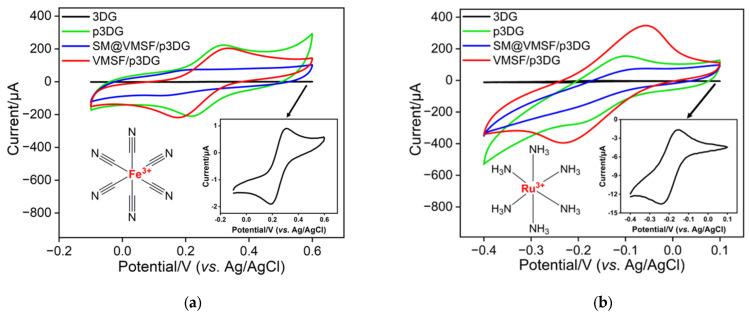
Cyclic voltametric curves obtained on 3DG, p3DG, SM@VMSF/p3DG, and VMSF/p3DG electrodes in (**a**) Fe(CN)_6_^3−^ (0.5 mM) or (**b**) Ru(NH_3_)_6_^3+^ (0.5 mM) solution (in 0.05 M KHP). Insets are illustrated structure of the redox probe (left) and the amplified curve obtained on 3DG.

Investigating the properties of different electrodes was also carried out by examining the CV curves of redox probes on various electrodes. The selected probes include the negatively charged probe Fe(CN)_6_^3−^ and the positively charged probe Ru(NH_3_)_6_^3+^. Figure 4a,b show the CV curves obtained on the 3DG, p3DG, micelle-containing SM@VMSF/p3DG, and VMSF/p3DG electrodes in the negatively charged Fe(CN)_6_^3−^ and positively charged Ru(NH_3_)_6_^3+^ probes. It can be observed that the 3DG electrode exhibits the lowest electrochemical signal response in both probe solutions, attributed to the strong hydrophobicity of 3DG, preventing the solution from infiltrating the working electrode surface and hindering direct electron transfer between the probe molecules and the working electrode. In contrast, the p3DG electrode obtained after electrochemical activation shows a higher signal response. This is due to the electrochemical polarization, which generates numerous active sites on the p3DG surface, enhancing its hydrophilicity. When VMSF grows on the electrode surface and SM is present in the nanochannels, the electrochemical signals of SM@VMSF/p3DG are weak in both redox probe solutions. This is because micelles block the nanochannels, preventing the probes from entering the channels and contacting the underlying electrode. Upon removal of SM, the signals of the VMSF/p3DG electrode increase in both probe solutions, indicating the opening of the nanochannels after SM removal. Compared to the p3DG electrode, the signals of the negatively charged probe Fe(CN)_6_^3−^ decrease, while the signals of the positively charged probe Ru(NH_3_)_6_^3+^ increase on the VMSF/p3DG electrode, demonstrating the charge-selective permeability of VMSF. This is attributed to the negatively charged surface. The silica structure of VMSF provides abundant silanol groups (p*K*_a_~2) on its surface, which ionize in the measured medium, repelling negatively charged probes and attracting positively charged probes.

### 3.3. Enhanced Electrochemical Signal of BHA on VMSF/p3DG Electrode

**Figure 5:**
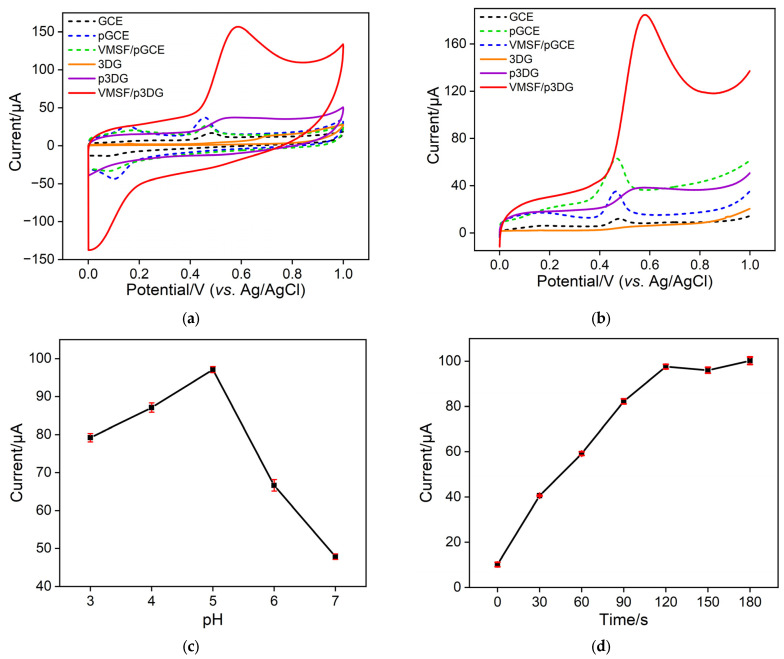
(**a**) Cyclic voltametric curves and (**b**) linear sweep voltametric curves obtained on different electrodes, including 3DG, p3DG, and VMSF/p3DG as well as 2D GCE, pGCE, and VMSF/pGCE in 0.1 M PBS (pH = 5.0) containing 20 μM BHA. Sweep speed was 0.1 V/s. The curves were obtained using the adsorption/enrichment time of 2 min. The adsorption enrichment process was carried out at the zero point. Oxidation peak current of BHA obtained using linear sweep voltammetry at different pH values of electrolyte (**c**) or different enrichment time (**d**).

To validate the feasibility of the prepared VMSF/p3DG sensor for BHA detection, electrochemical signals of BHA on the different electrodes were compared. The examined electrodes included 3DG, p3DG, and VMSF/p3DG. Figure 5a,b displays the CV and LSV curves of BHA on these electrodes. For the 3DG electrode (orange solid line), the typical voltammogram, similar to that previously reported, was observed [64,65]. However, very low peak current signals for BHA were observed, attributed to the high hydrophobicity of 3DG. When the 3DG electrode was activated to form a p3DG electrode (purple solid line), the introduction of numerous oxygen-containing functional groups during activation enhanced its hydrophilicity, along with an increase in its specific surface area. Consequently, p3DG exhibited a significantly improved signal response to BHA. Additionally, the oxidation peak potential of BHA on p3DG (0.6 V) was lower than that on 3DG (0.7 V), indicating the electrocatalytic effect of oxygen-containing groups or defect sites formed on p3DG through electrochemical activation. When a VMSF layer was prepared on the electrode surface to form the VMSF/p3DG electrode (red solid line), the oxidation peak current measured by LSV was 129.7 μA, with a relative standard deviation (RSD) for the three measurements of 2.9%, approximately 8.5 times higher than that on p3DG. As the insulating property of silica-based VSMF would reduce the surface area of p3DG, the enhanced signal indicated that the VMSF nanochannels have a strong enrichment effect on BHA.

As a control, the signals of BHA were compared on a commonly used two-dimensional carbon electrode, GCE, and an electrode obtained by the same electrochemical activation (pGCE), as well as a film-modified electrode (VMSF/pGCE). It can be observed that the signal of BHA on the GCE electrode (black dashed line) exhibited only a small peak current. When the GCE electrode was pre-activated to form a pGCE electrode (blue dashed line), the peak response of BHA increased. This is attributed to the increase in the electrode’s electroactive surface area after electrochemical activation. When the film was grown on the pGCE surface to obtain VMSF/pGCE (green dashed line), the detection signal for BHA slightly weakened. This may be due to the fact that, for a 2D planar electrode, the insulating effect of VMSF on the surface area of pGCE is stronger than the enrichment effect of VMSF nanochannels. These results confirm that integrating VMSF into a 3D electrode can enhance the signal sensitivity for BHA, demonstrating advantages in highly sensitive electrochemical detection of BHA.

### 3.4. Optimization of BHA Detection Conditions

To enhance detection performance, the optimization of detection conditions, including electrolyte pH and BHA enrichment time, was carried out. In Figure 5c, the signal response of BHA on VMSF/p3DG was observed across the pH range of 3 to 7. Notably, the VMSF/p3DG electrode demonstrated the highest current signal for BHA at pH 5. The LSV curves of BHA on the VMSF/p3DG electrodes were determined under different pH conditions. As shown in Figure S3 (Supporting Information), the oxidation peak potential (*E*pa) of BHA shifted negatively with an increase in pH. There exists a linear relationship between *E*pa and pH (*E*pa = −0.0674pH + 0.918). The following formula was employed to calculate the number of protons (*m*) and electrons (*n*) involved in the electrochemical process of BHA:

$$\frac{{dE}_{pa}}{dpH}=2.303\frac{mRT}{nF}$$

where F is the Faraday constant (96,485 C·mol^−1^), *R* is the molar gas constant (8.314 J·mol^−1^·K^−1^), and T is the absolute temperature. The calculated m/n ratio for the oxidation process of BHA is 1.2, close to 1, indicating that the number of protons and the number electrons involved in the oxidation of BHA are equal. This suggests a process with equal electrons and protons. The structural changes during the electrochemical oxidation process of BHA are illustrated as follows [64,65]:


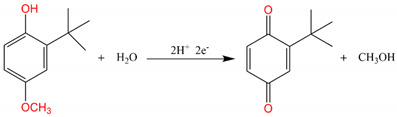


As seen in Figure 5d, an increase in enrichment time led to a gradual rise in the LSV oxidation peak current of BHA, reaching a plateau at 120 s of enrichment time. Therefore, pH 5 and an enrichment time of 120 s were identified as the optimal detection conditions.

### 3.5. Electrochemical Detection of BHA

**Figure 6:**
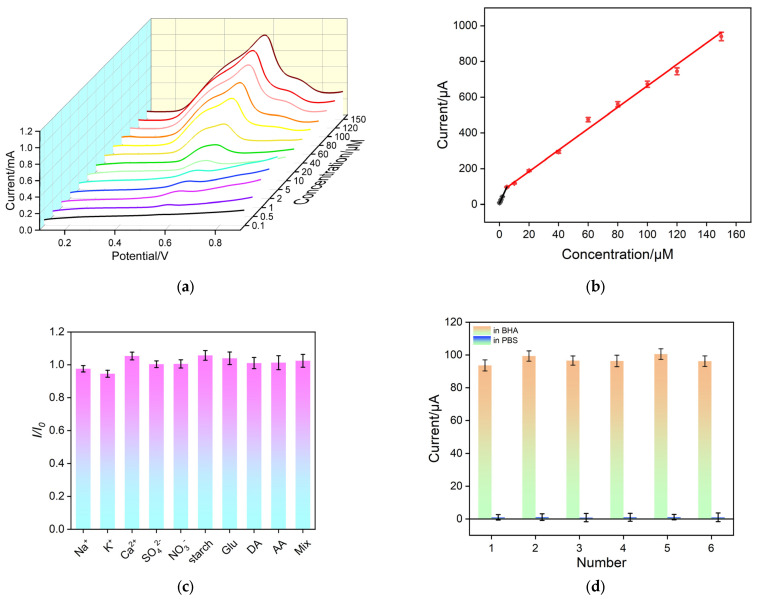
(**a**) Linear sweep voltametric curves obtained on VMSF/p3DG electrode in PBS (0.1 M, pH = 5) containing different concentrations of BHA. (**b**) Relationship of anodic peak current in linear sweep voltametric curves with BHA concentration. (**c**) Anodic peak current obtained on linear sweep voltametric curves using VMSF/p3DG electrode in the absence (*I*_0_) or presence (*I*) of different interferents. The concentration of BHA is 5 μM. The concentration of the other substance is 10 μM. (**d**) Anodic peak current obtained on linear sweep voltametric curves using regenerated VMSF/p3DG electrode in BHA (5 μM) or PBS.

Under the optimal conditions, electrochemical measurements to assess the detection performance of the VMSF/p3DG sensor with regard to BHA was investigated. In Figure 6a, LSV curves on the VMSF/p3DG electrode for different concentrations of BHA in PBS electrolyte solution are presented. As shown, the oxidation peak current (*I*) gradually increases with the increase in BHA concentration (*C*), exhibiting two well-correlated curves (Figure 6b). The linear detection range is determined to be 0.1 μM to 5 μM (*I* = 19.2 (±0.674) C + 1.79 (±1.87), *R*^2^ = 0.998) and 5 μM to 150 μM (*I* = 5.99 (±0.180) C + 64.1 (±4.56), *R*^2^ = 0.992). The limit of detection (LOD) calculated using three signal-to-noise ratios (S/N = 3) is determined to be 12 nM. The limit of quantification is 39.6 nM based on ten signal-to-noise ratios (S/N = 10). The analytical performance of various methods for the detection of CEA is summarized in Table S1 (Supporting Information) [65,66,67,68,69,70,71,72,73,74]. The LOD is lower than that obtained using the gold nanoparticle/electrochemical reduced graphene oxide-modified glass carbon electrode (AuNPs/ERGO/GCE) [66], multi-walled carbon nanotube-modified screen-printed electrode (MWCNT/SPE) [67], multi-walled carbon nanotube/poly O-cresolphthalein complexone-modified paraffin wax-impregnated graphite electrode (MWCNTs/POC/PIG) [68], graphene/vholine-modified GCE (Graphene/Ch/GCE) [65], poly copper (II) phosphate-modified carbon composite electrode (MCCE-Cu_3_(PO_4_)^2−^-Poly) [69], ZnO spheres@graphene oxide nanosheets-modified GCE (Zn TPHS@GO/GCE) [70], and lithium tetracyanoethylenide/titanium dioxide-modified indium tin oxide electrode (LiTCNE/TiO_2_/ITO) [71], but higher than that obtained on the molecularly imprinted polymer/molybdenum disulfide/Ag nanoparticle-chitosan-modified GCE (MIP/MoS_2_/Ag NPs-CS/GCE) [72]. The LOD is also higher than that obtained using solid phase extraction and gas chromatography/mass spectrometry (SPE-GC/MS, EPA/600/R-14/442, USA), the two-step microextraction-based GC/MS [73], or the covalent organic framework (COF)-based monolithic column solid-phase microextraction-HPLC system (SPME-HPLC) [74].

### 3.6. Anti-Interference/Anti-Fouling and Regeneration Characteristics of VMSF/p3DG Sensor

To meet the practical application requirements of the sensor, the anti-interference and anti-fouling capabilities of the VMSF/p3DG electrode were investigated. As shown in Figure 6c, some substances that might coexist in food samples, including inorganic ions (Na^+^, K^+^, Ca^2+^, SO_4_^2−^), starch, glucose (Glu), and vitamins (ascorbic acid (AA)) were selected as interferents. In addition, the effect of the common redox molecule, uric acid (UA), was also investigated. The ratio of the LSV oxidation peak current values with the absence of interferents (*I*_0_) to the presence of interferents (*I*) at equivalent concentrations was calculated. The results indicate that the addition of interferent ions at twice the concentration of that of BHA did not significantly alter the current, demonstrating the excellent selectivity and anti-interference/anti-fouling capability of the VMSF/p3DG electrode. This is attributed to the size exclusion effect provided by VMSF, preventing large molecules from reaching the electrode surface through the nanopores, thus preventing fouling. In addition, the potential resolution of p3DG also contributed to the selectivity.

Additionally, to evaluate the regeneration capability of the electrode, it was immersed in a 0.1 M HCl aqueous solution to eliminate residual substances from the nanochannels after detection. Subsequently, the regenerated electrode was employed for another round of electrochemical testing. As depicted in Figure 6d, the regenerated electrode showed a nearly negligible oxidation current in the electrolyte, indicating the thorough removal of BHA from the electrode. Following six regeneration cycles, the detected signal values stabilized at 94% to 101% of the initial signal, showcasing outstanding regeneration performance.

### 3.7. Real Sample Analysis

To investigate the potential of the developed VMSF/p3DG sensor in real sample analysis, BHA in edible oil and coffee samples was detected using the standard addition method (Figures S4 and S5 in Supporting Information). The content of BHA (5.49 μM) in the edible oil (100 mg/mL) was lower than the maximum allowable concentration (55.6 μM for 100 mg/mL edible oil sample, calculated based on 100 mg/kg), while BHA was not detected in the coffee. As shown in Table 1, satisfactory recovery rates obtained using the standard addition method ranging from 97.9% to 103% were revealed, with a small relative standard deviation (RSD, <3.8%), indicating high reliability. Owing to the simple fabrication of the sensor and simple detection without tedious sample pre-treatment, the fabricated sensor is promising for the determination of BHA content in complex food samples.

## 4. Conclusions

In this work, a three-dimensional sensor was easily fabricated based on integration of VMSF with a 3D graphene electrode, which enabled sensitive electrochemical detection of BHA in food. The 3DG electrode was surface-activated through simple electrochemical polarization, effectively improving its hydrophilicity and electrochemical activity. Using the p3DG electrode as the supporting electrode, a stable VMSF layer could be rapidly grown on its surface through the EASA method, without the need for an additional adhesive layer. Due to the enrichment effect of nanochannels, the VMSF/p3DG electrode exhibited high sensitivity in detecting BHA. Combining the excellent fouling resistance and anti-interference properties of VMSF, the VMSF/p3DG electrode can be applied for rapid and highly sensitive detection of BHA in food samples. This work achieves the integration of VMSF with a three-dimensional electrode, providing a new approach for developing high-performance VMSF-based electrochemical sensors and analyzing antioxidants in complex food samples.

## Figures and Tables

**Table 1:** Determination of BHA in food samples.

Sample ^a^	Added (μM)	Found (μM)	Recovery (%)	RSD (%, n = 3)
Edible oil	0.00	5.49	-	3.8
	5.00	10.62	103	3.2
	10.0	15.38	98.9	1.8
	50.0	56.02	101	2.1
Coffee ^b^	5.00	5.06	101	3.7
	10.0	9.79	97.9	3.0
	50.0	50.3	101	1.1

^a^ Edible oil samples were 100 mg/mL in ethanol. Coffee samples were aqueous solution (100 mg/mL). All samples before and after BHA spiking were diluted with PBS (0.1 M, pH = 5) 50 times before measurement. The indicated concentration was obtained before dilution. ^b^ BHA was not detected in the coffee samples.

## Data Availability

The data presented in this study are available on request from the corresponding author.

## Supplementary Materials

The following supporting information can be downloaded at: https://www.mdpi.com/article/10.3390/nano14070569/s1, Figure S1. SEM image of VMSF/p3DG (b). Figure S2. The element content of 3DG (a) or p3DG (b) obtained from XPS data. Figure S3. (a) Baseline-corrected linear sweep voltammetry curves of BHA at different pH in 0.1 M PBS. (b) Linear regression curve between the plots of anodic peak potential and pH values. Figure S4. (a) Baseline-corrected linear sweep voltammetry curves obtained on edible oil samples in absence or presence of spiked BHA. (b) The linear extrapolation curve obtained using the standard addition method for the detection of BHA in edible oil samples. All samples before and after BHA spiking were diluted with PBS (0.1 M, pH = 5) for 50 times before measurement. The indicated concentration was obtained after dilution. Figure S5. (a) Baseline-corrected linear sweep voltammetry curves obtained on coffee samples in absence or presence of spiked BHA. (b) The linear extrapolation curve obtained using the standard addition method for the detection of BHA in coffee samples. All samples before and after BHA spiking were diluted with PBS (0.1 M, pH = 5) for 50 times before measurement. The indicated concentration was obtained after dilution. Table S1. Comparison of the analytical performance of different methods for BHA detection.

## References

[1] Ma G., Wang Y., Li Y., Zhang L., Gao Y., Li Q., Yu X. (2023). Antioxidant properties of lipid concomitants in edible oils: A review. Food Chem..

[2] Carocho M., Morales P., Ferreira I.C. (2018). Antioxidants: Reviewing the chemistry, food applications, legislation and role as preservatives. Trends Food Sci. Technol..

[3] Gutiérrez-del-Río I., López-Ibáñez S., Magadán-Corpas P., Fernández-Calleja L., Pérez-Valero Á., Tuñón-Granda M., Miguélez E.M., Villar C.J., Lombó F. (2021). Terpenoids and polyphenols as natural antioxidant agents in food preservation. Antioxidants.

[4] André C., Castanheira I., Cruz J.M., Paseiro P., Sanches-Silva A. (2010). Analytical strategies to evaluate antioxidants in food: A review. Trends Food Sci. Technol..

[5] Motia S., Bouchikhi B., Bari N.E. (2021). An electrochemical molecularly imprinted sensor based on chitosan capped with gold nanoparticles and its application for highly sensitive butylated hydroxyanisole analysis in foodstuff products. Talanta.

[6] Saad B., Sing Y., Nawi M., Hashim N., Mohamedali A., Saleh M., Sulaiman S., Talib K., Ahmad K. (2007). Determination of synthetic phenolic antioxidants in food items using reversed-phase HPLC. Food Chem..

[7] Guo L., Xie M., Yan A., Wan Y., Wu Y. (2006). Simultaneous determination of five synthetic antioxidants in edible vegetable oil by GC-MS. Anal. Bioanal. Chem..

[8] Munteanu I.G., Apetrei C. (2022). A Review on electrochemical sensors and biosensors used in assessing antioxidant activity. Antioxidants.

[9] Zhang Y., Lei Y., Lu H., Shi L., Wang P., Ali Z., Li J. (2021). Electrochemical detection of bisphenols in food: A review. Food Chem..

[10] Wang K., Lin X., Zhang M., Li Y., Luo C., Wu J. (2022). Review of electrochemical biosensors for food safety detection. Biosensors.

[11] He J., Xu X., Li M., Zhou S., Zhou W. (2023). Recent advances in perovskite oxides for non-enzymatic electrochemical sensors: A review. Anal. Chim. Acta.

[12] He J., Xu X., Sun H., Miao T., Li M., Zhou S., Zhou W. (2023). Participation of lattice oxygen in perovskite oxide as a highly sensitive sensor for p-phenylenediamine detection. Molecules.

[13] Ng K.L., Tan G.H., Khor S.M. (2017). Graphite nanocomposites sensor for multiplex detection of antioxidants in food. Food Chem..

[14] Zhu X., Xuan L., Gong J., Liu J., Wang X., Xi F., Chen J. (2022). Three-dimensional macroscopic graphene supported vertically-ordered mesoporous silica-nanochannel film for direct and ultrasensitive detection of uric acid in serum. Talanta.

[15] Deng X., Lin X., Zhou H., Liu J., Tang H. (2023). Equipment of vertically-ordered mesoporous silica film on electrochemically pretreated three-dimensional graphene electrodes for sensitive detection of methidazine in urine. Nanomaterials.

[16] Han J., Johnson I., Chen M. (2022). 3D Continuously porous graphene for energy applications. Adv. Mater..

[17] Zhou H., Dong G., Sailjoi A., Liu J. (2022). Facile pretreatment of three-dimensional graphene through electrochemical polarization for improved electrocatalytic performance and simultaneous electrochemical detection of catechol and hydroquinone. Nanomaterials.

[18] Zheng W., Su R., Lin X., Liu J. (2022). Nanochannel array modified three-dimensional graphene electrode for sensitive electrochemical detection of 2,4,6-trichlorophenol and prochloraz. Front. Chem..

[19] Gong J., Tang H., Wang M., Lin X., Wang K., Liu J. (2022). Novel three-dimensional graphene nanomesh prepared by facile electro-etching for improved electroanalytical performance for small biomolecules. Mater. Des..

[20] Ullah S., Hasan M., Ta H.Q., Zhao L., Shi Q., Fu L., Choi J., Yang R., Liu Z., Rümmeli M.H. (2019). Synthesis of doped porous 3D graphenestructures by chemical vapor deposition and its applications. Adv. Funct. Mater..

[21] Khan A., Habib M.R., Kumar R.R., Islam S.M., Arivazhagan V., Salman M., Yang D., Yu X. (2018). Wetting behaviors and applications of metal-catalyzed CVD grown graphene. J. Mater. Chem. A.

[22] Xiao W., Li B., Yan J., Wang L., Huang X., Gao J. (2023). Three dimensional graphene composites: Preparation, morphology and their multi-functional applications. Compos. Part A Appl. Sci. Manuf..

[23] Liu Q., Zhong H., Chen M., Zhao C., Liu Y., Xi F., Luo T. (2020). Functional nanostructure-loaded three-dimensional graphene foam as a non-enzymatic electrochemical sensor for reagentless glucose detection. RSC Adv..

[24] Rezvani E., Hatamie A., Berahman M., Simchi M., Angizi S., Rahmati R., Kennedy J., Simchi A. (2019). Synthesis, first-principle simulation, and application of three-dimensional ceria nanoparticles/graphene nanocomposite for non-enzymatic hydrogen peroxide detection. J. Electrochem. Soc..

[25] Xi F., Zhao D., Wang X., Chen P. (2013). Non-enzymatic detection of hydrogen peroxide using a functionalized three-dimensional graphene electrode. Electrochem. Commun..

[26] Feng X., Zhang Y., Zhou J., Li Y., Chen S., Zhang L., Ma Y., Wang L., Yan X. (2015). Three-dimensional nitrogen-doped graphene as an ultrasensitive electrochemical sensor for the detection of dopamine. Nanoscale.

[27] Liu J., Wang J., Wang T., Li D., Xi F., Wang J., Wang E. (2015). Three-dimensional electrochemical immunosensor for sensitive detection of carcinoembryonic antigen based on monolithic and macroporous graphene foam. Biosens. Bioelectron..

[28] Wu X., Wang K., Zhang J., Jie X., Chen Z., Lai Y. (2022). A polyester-silica anti-condensation surface with anti-fouling property. Chem. Eng. J..

[29] Qi L., Liang R., Jiang T., Qin W. (2022). Anti-fouling polymeric membrane ion-selective electrodes. Trends Anal. Chem..

[30] Wang K., Yang L., Huang H., Lv N., Liu J., Liu Y. (2022). Nanochannel array on electrochemically polarized screen printed carbon electrode for rapid and sensitive electrochemical determination of clozapine in human whole blood. Molecules.

[31] Zhang C., Zhou X., Yan F., Lin J. (2023). N-Doped Graphene quantum dots confined within silica nanochannels for enhanced electrochemical detection of doxorubicin. Molecules.

[32] Li Y., Wu Q., Wu Z., Zhuang Y., Sun L., Fan X., Zhao T., Yi L., Gu Y. (2023). Biomimetic functional material-based sensors for food safety analysis: A review. Food Chem..

[33] Zhang T., Xu S., Lin X., Liu J., Wang K. (2023). Label-free electrochemical aptasensor based on the vertically-aligned mesoporous silica films for determination of aflatoxin B1. Biosensors.

[34] Ma N., Xu S., Wu W., Liu J. (2023). Electrochemiluminescence aptasensor with dual signal amplification by silica nanochannel-based confinement effect on nanocatalyst and efficient emitter enrichment for highly sensitive detection of C-reactive protein. Molecules.

[35] Huang J., Xu S., Yan F., Liu J. (2024). Electrochemiluminescence enzyme biosensors for ultrasensitive determination of glucose using glucose dehydrogenase immobilized on vertical silica nanochannels. Sens. Actuators B Chem..

[36] Villa C.C., Galus S., Nowacka M., Magri A., Petriccione M., Gutiérrez T.J. (2020). Molecular sieves for food applications: A review. Trends Food Sci. Technol..

[37] Ma K., Yang L., Liu J., Liu J. (2022). Electrochemical sensor nanoarchitectonics for sensitive detection of uric acid in human whole blood based on screen-printed carbon electrode equipped with vertically-ordered mesoporous silica-nanochannel film. Nanomaterials.

[38] Luo X., Zhang T., Tang H., Liu J. (2022). Novel electrochemical and electrochemiluminescence dual-modality sensing platform for sensitive determination of antimicrobial peptides based on probe encapsulated liposome and nanochannel array electrode. Front. Nutr..

[39] Huang J., Zhang T., Zheng Y., Liu J. (2023). Dual-mode sensing platform for cancer antigen 15-3 determination based on a silica nanochannel array using electrochemiluminescence and electrochemistry. Biosensors.

[40] Chen D., Luo X., Xi F. (2023). Probe-integrated electrochemical immunosensor based on electrostatic nanocage array for reagentless and sensitive detection of tumor biomarker. Front. Chem..

[41] Chang Q., Gu X., He L., Xi F. (2023). A highly sensitive immunosensor based on nanochannel-confined nano-gold enhanced electrochemiluminescence for procalcitonin detection. Front. Chem..

[42] Zhou X., Han Q., Zhou J., Liu C., Liu J. (2023). Reagentless electrochemical detection of tumor biomarker based on stable confinement of electrochemical probe in bipolar silica nanochannel film. Nanomaterials.

[43] Teng Z., Zheng G., Dou Y., Li W., Mou C.Y., Zhang X., Asiri A.M., Zhao D. (2012). Highly ordered mesoporous silica films with perpendicular mesochannels by a simple stöber-solution growth approach. Angew. Chem. Int. Ed..

[44] Zhang H., Zhang C., Qu H., Xi F. (2023). Immunosensor with enhanced electrochemiluminescence signal using platinum nanoparticles confined within nanochannels for highly sensitive detection of carcinoembryonic antigen. Molecules.

[45] Walcarius A. (2021). Electroinduced surfactant self-assembly driven to vertical growth of oriented mesoporous films. Acc. Chem. Res..

[46] Zhou X., Gu X., Zhang S., Zou Y., Yan F. (2024). Magnetic graphene oxide and vertically-ordered mesoporous silica film for universal and sensitive homogeneous electrochemiluminescence aptasensor platform. Microchem. J..

[47] Li F., Han Q., Xi F. (2023). The fabrication of a probe-integrated electrochemiluminescence aptasensor based on double-layered nanochannel array with opposite charges for the sensitive determination of C-reactive protein. Molecules.

[48] Zhou H., Ding Y., Su R., Lu D., Tang H., Xi F. (2022). Silica nanochannel array film supported by ß-cyclodextrin-functionalized graphene modified gold film electrode for sensitive and direct electroanalysis of acetaminophen. Front. Chem..

[49] Yu R., Zhao Y., Liu J. (2024). Solid electrochemiluminescence sensor by immobilization of emitter ruthenium(ii)tris(bipyridine) in bipolar silica nanochannel film for sensitive detection of oxalate in serum and urine. Nanomaterials.

[50] Xing J., Han Q., Liu J., Yan Z. (2023). Electrochemical aptasensor fabricated by anchoring recognition aptamers and immobilizing redox probes on bipolar silica nanochannel array for reagentless detection of carbohydrate antigen 15-3. Front. Chem..

[51] Wang Q., Yang Q., Su B. (2015). Adsorption of microperoxidase-11 in vertical silica mesochannels and electrochemical investigation of its electron transfer properties. Electrochim. Acta.

[52] Su R., Tang H., Xi F. (2022). Sensitive electrochemical detection of p-nitrophenol by pre-activated glassy carbon electrode integrated with silica nanochannel array film. Front. Chem..

[53] Lv N., Qiu X., Han Q., Xi F., Wang Y., Chen J. (2022). Anti-biofouling electrochemical sensor based on the binary nanocomposite of silica nanochannel array and graphene for doxorubicin detection in human serum and urine samples. Molecules.

[54] Huang L., Su R., Xi F. (2023). Sensitive detection of noradrenaline in human whole blood based on Au nanoparticles embedded vertically-ordered silica nanochannels modified pre-activated glassy carbon electrodes. Front. Chem..

[55] Zhou L., Ding H., Yan F., Guo W., Su B. (2018). Electrochemical detection of Alzheimer’s disease related substances in biofluids by silica nanochannel membrane modified glassy carbon electrodes. Analyst.

[56] Gong J., Tang H., Luo X., Zhou H., Lin X., Wang K., Fei Y., Xi F., Liu J. (2021). Vertically ordered mesoporous silica-nanochannel film-equipped three-dimensional macroporous graphene as sensitive electrochemiluminescence platform. Front. Chem..

[57] Xi F., Xuan L., Lu L., Huang J., Yan F., Liu J., Dong X., Chen P. (2019). Improved adhesion and performance of vertically-aligned mesoporous silica-nanochannel film on reduced graphene oxide for direct electrochemical analysis of human serum. Sens. Actuators B Chem..

[58] Ananthanarayanan A., Wang X., Routh P., Sana B., Lim S., Kim D., Lim K., Li J., Chen P. (2014). Facile synthesis of graphene quantum dots from 3D graphene and their Application for Fe^3+^ Sensing. Adv. Funct. Mater..

[59] Walcarius A., Sibottier E., Etienne M., Ghanbaja J. (2007). Electrochemically assisted self-assembly of mesoporous silica thin films. Nat. Mater..

[60] Wang L., Yang R., Wang H., Li J., Qu L., Harrington P.d.B. (2015). High-selective and sensitive voltammetric sensor for butylated hydroxyanisole based on AuNPs-PVP-graphene nanocomposites. Talanta.

[61] Sousa Carvalho R.M., Yotsumoto Neto S., Carvalho Silva F., Santos Damos F., de Cássia Silva Luz R. (2016). A sensitive sensor based on CuTSPc and reduced graphene oxide for simultaneous determination of the BHA and TBHQ antioxidants in biodiesel samples. Electroanalysis.

[62] Yong Y., Dong X., Chan-Park M.B., Song H., Chen P. (2012). Macroporous and monolithic anode based on polyaniline hybridized three-dimensional graphene for high-performance microbial fuel cells. ACS Nano.

[63] Qiu H., Guan Y., Luo P., Wang Y. (2017). Recent advance in fabricating monolithic 3D porous graphene and their applications in biosensing and biofuel cells. Biosens. Bioelectron..

[64] Fan L., Kan X. (2020). Sensitive detection of butylated hydroxyanisole based on free-standing paper decorated with gold and NiO nanoparticles. Microchem. J..

[65] Wang P., Han C., Zhou F., Lu J., Han X., Wang Z. (2016). Electrochemical determination of tert-butylhydroquinone and butylated hydroxyanisole at choline functionalized film supported graphene interface. Sens. Actuators B Chem..

[66] Yue X., Song W., Zhu W., Wang J., Wang Y. (2015). In situ surface electrochemical co-reduction route towards controllable construction of AuNPs/ERGO electrochemical sensing platform for simultaneous determination of BHA and TBHQ. Electrochim. Acta.

[67] Caramit R.P., de Freitas Andrade A.G., Gomes de Souza J.B., de Araujo T.A., Viana L.H., Trindade M.A.G., Ferreira V.S. (2013). A new voltammetric method for the simultaneous determination of the antioxidants TBHQ and BHA in biodiesel using multi-walled carbon nanotube screen-printed electrodes. Fuel.

[68] Manoranjitham J.J., Narayanan S.S. (2021). Electrochemical sensor for determination of butylated hydroxyanisole (BHA) in food products using poly O-cresolphthalein complexone coated multiwalled carbon nanotubes electrode. Food Chem..

[69] Freitas K., Fatibello-Filho O. (2010). Simultaneous determination of butylated hydroxyanisole (BHA) and butylated hydroxytoluene (BHT) in food samples using a carbon composite electrode modified with Cu_3_(PO_4_)_2_ immobilized in polyester resin. Talanta.

[70] Gan T., Zhao A., Wang S., Lv Z., Sun J. (2016). Hierarchical triple-shelled porous hollow zinc oxide spheres wrapped in graphene oxide as efficient sensor material for simultaneous electrochemical determination of synthetic antioxidants in vegetable oil. Sens. Actuators B Chem..

[71] Monteiro T., Yotsumoto Neto S., Damos F., Luz R. (2016). Development of a photoelectrochemical sensor for detection of TBHQ antioxidant based on LiTCNE-TiO_2_ composite under visible LED light. J. Electroanal. Chem..

[72] Han S., Ding Y., Teng F., Yao A., Leng Q. (2022). Molecularly imprinted electrochemical sensor based on 3D-flower-like MoS2 decorated with silver nanoparticles for highly selective detection of butylated hydroxyanisole. Food Chem..

[73] Ding M., Zou J. (2012). Rapid micropreparation procedure for the gas chromatographic-mass spectrometric determination of BHT, BHA and TBHQ in edible oils. Food Chem..

[74] Wang J., Hao Y., Ni B., Sun J., Wu X., Lin X. (2023). Covalent organic framework-based monolithic column with hydrophilic and π-π stacking interaction for efficient in-tube solid-phase microextraction of synthetic phenolic antioxidants. Microchem. J..

