# Supplementary material for: Highly Sensitive Electrochemical Determination of Butylated Hydroxyanisole in Food Samples Using Electrochemical-Pretreated Three-Dimensional Graphene Electrode Modified with Silica Nanochannel Film
Source: Nanomaterials (Basel). 2024 Mar 25;14(7):569. doi: 10.3390/nano14070569 (PMC11013248; doi:10.3390/nano14070569)
Supplement: Supplementary file 1 [file nanomaterials-14-00569-s001.zip › nanomaterials-2857158-supplementary.pdf]

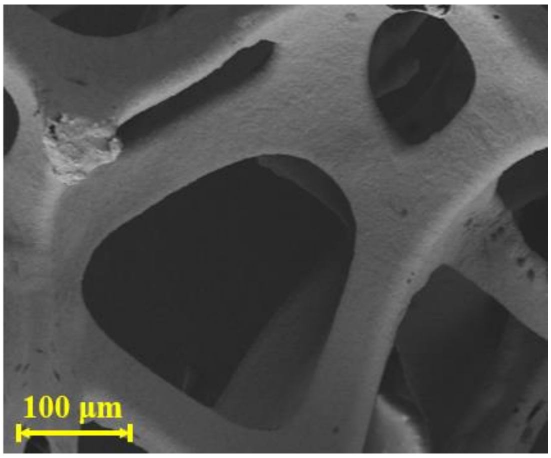

Figure S1. SEM image of VMSF/p3DG (b).

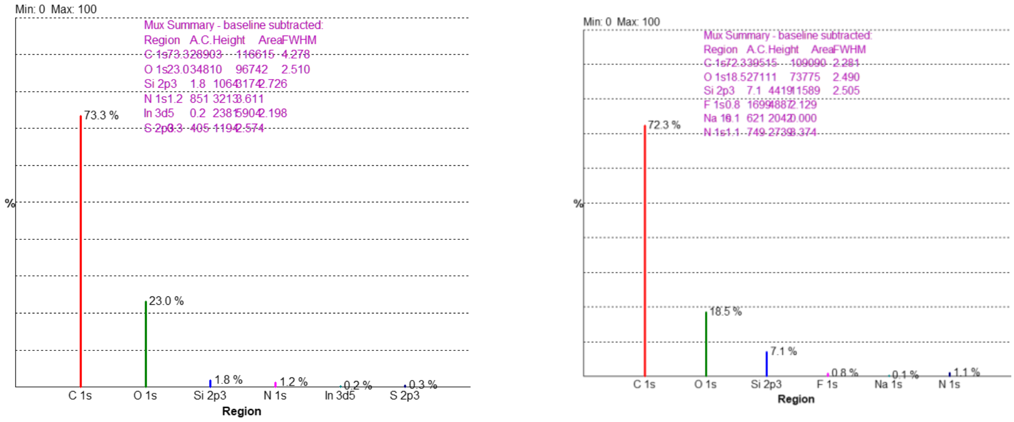

(a) (b)

Figure S2. The element content of 3DG (a) or p3DG (b) obtained from XPS data.

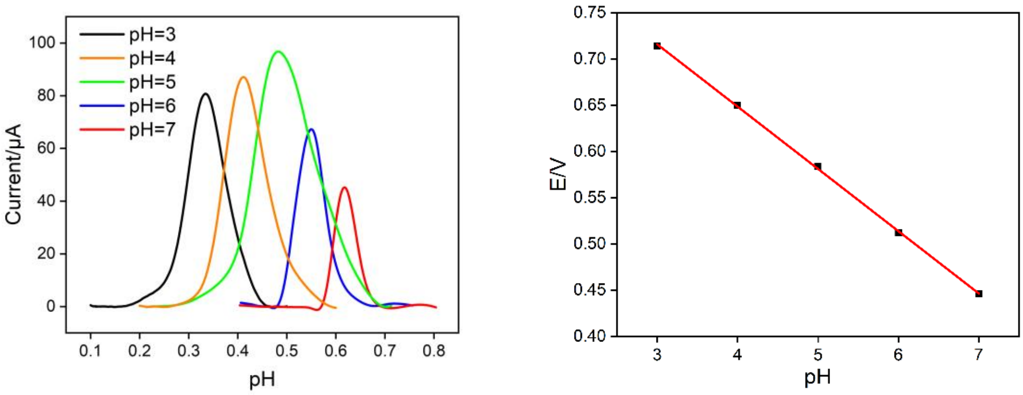

(a) (b)

Figure S3. (a) Baseline-corrected linear sweep voltammetry curves of BHA at different pH in 0.1 M PBS. (b) Linear regression curve between the plots of anodic peak potential and pH values.

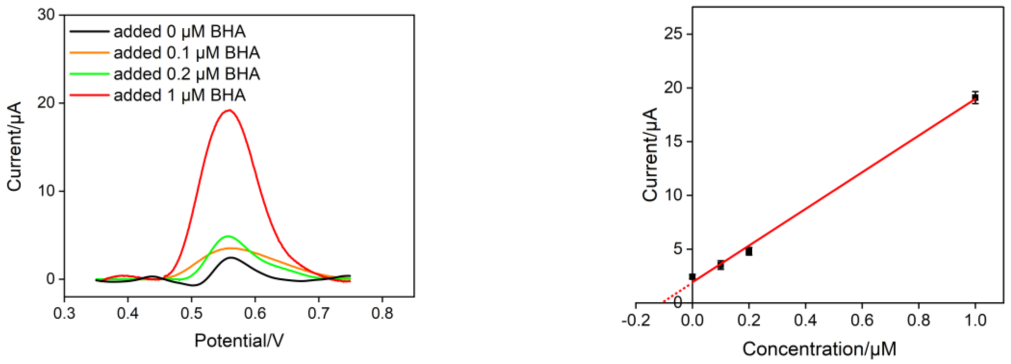

(a) (b)

Figure S4. (a) Baseline-corrected linear sweep voltammetry curves obtained on edible oil samples in absence or presence of spiked BHA. (b) The linear extrapolation curve obtained using the standard addition method for the detection of BHA in edible oil samples. All samples before and after BHA spiking were diluted with PBS (0.1 M, pH=5) for 50 times before measurement. The indicated concentration was obtained after dilution.

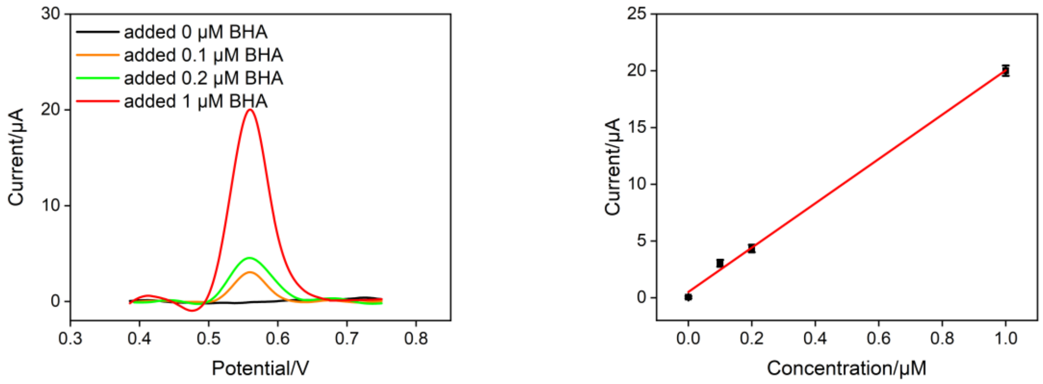

(a) (b)

Figure S5. (a) Baseline-corrected linear sweep voltammetry curves obtained on coffee samples in absence or presence of spiked BHA. (b) The linear extrapolation curve obtained using the standard addition method for the detection of BHA in coffee samples. All samples before and after BHA spiking were diluted with PBS (0.1 M, pH=5) for 50 times before measurement. The indicated concentration was obtained after dilution.

Table S1. Comparison of the analytical performance of different methods for BHA detection.

Electrode	Detection method	Linear range (μM)	LOD (μM)	Reference
AuNPs/ERGO/GCE	LSV	0.56 - 55.6	0.23	[66]
MWCNT/SPE	LSV	0.5 - 10	1.14	[67]
MWCNTs/POC/PIG	DPV	0.33 - 110	0.11	[68]
Graphene/Ch/GCE	DPV	0.60 - 200	0.19	[65]
MIP/MoS_2/Ag NPs-CS/GCE	DPV	0.001 - 100	0.0079	[69]
MCCE–$Cu_3(PO_4)^{2-}$-Poly	SWV	0.34 - 41	0.072	[70]
Zn TPHS@GO/GCE	SWV	0.30 - 60	48.8	[71]
LiTCNE/TiO_2/ITO	ECL	0.4 - 500	0.10	[72]
two-step microextraction based GC/MS	GC/MS	0.055 – 110	0.011	[73]
SPME-HPLC	HPLC-UV	0.028 – 16.6	0.0011	[74]
VMSF/p3DG	LSV	0.1 - 5, 5 - 150	0.012	**This work**

AuNPs: Gold nanoparticles; ERGO: electrochemical reduced graphene oxide; GCE: glass carbon electrode; SPE: screen-printed electrodes; MWCNT: multi-walled carbon nanotube; POC: poly O-cresolphthalein complexone; PIG: paraffin wax impregnated graphite electrode; DPV: differential pulse voltammetry; Ch: Choline; MIP: molecularly imprinted polymer; MoS_2: molybdenum disulfide; Ag NPs-CS: Ag nanoparticle-chitosan; MCCE: modified carbon composite electrode; $Cu_3(PO_4)^{2-}$-Poly: poly copper (II) phosphate; SWV: square wave voltammetry; Zn TPHS@GO: ZnO spheres@graphene oxide nanosheets; LiTCNE: lithium tetracyanoethylenide; TiO_2: titanium dioxide; ECL: electrochemiluminescence; GC/MS: gas chromatography/mass spectrometry; SPME-HPLC: solid-phase microextraction-high performance liquid chromatography.
